# Opportunities for Conventional and In Situ Cancer Vaccine Strategies and Combination with Immunotherapy for Gastrointestinal Cancers, A Review

**DOI:** 10.3390/cancers12051121

**Published:** 2020-04-30

**Authors:** Rachid Bouzid, Maikel Peppelenbosch, Sonja I. Buschow

**Affiliations:** Department of Gastroenterology and Hepatology, Erasmus MC, 3015GD Rotterdam, The Netherlands; r.bouzid@erasmusmc.nl (R.B.); m.peppelenbosch@erasmusmc.nl (M.P.)

**Keywords:** cancer vaccines, in situ vaccination, immunotherapy

## Abstract

Survival of gastrointestinal cancer remains dismal, especially for metastasized disease. For various cancers, especially melanoma and lung cancer, immunotherapy has been proven to confer survival benefits, but results for gastrointestinal cancer have been disappointing. Hence, there is substantial interest in exploring the usefulness of adaptive immune system education with respect to anti-cancer responses though vaccination. Encouragingly, even fairly non-specific approaches to vaccination and immune system stimulation, involving for instance influenza vaccines, have shown promising results, eliciting hopes that selection of specific antigens for vaccination may prove useful for at least a subset of gastrointestinal cancers. It is widely recognized that immune recognition and initiation of responses are hampered by a lack of T cell help, or by suppressive cancer-associated factors. In this review we will discuss the hurdles that limit efficacy of conventional cancer therapeutic vaccination methods (e.g., peptide vaccines, dendritic cell vaccination). In addition, we will outline other forms of treatment (e.g., radiotherapy, chemotherapy, oncolytic viruses) that also cause the release of antigens through immunogenic tumor cell death and can thus be considered unconventional vaccination methods (i.e., in situ vaccination). Finally, we focus on the potential additive value that vaccination strategies may have for improving the effect immunotherapy. Overall, a picture will emerge that although the field has made substantial progress, successful immunotherapy through the combination with cancer antigen vaccination, including that for gastrointestinal cancers, is still in its infancy, prompting further intensification of the research effort in this respect.

## 1. Introduction

Clinical management of oncological disease of the gastrointestinal tract remains very challenging especially when surgical options have been exhausted. The problem gastrointestinal cancer poses for medicine and society at large is compounded by the nosidynamics of this group of diseases, for many gastrointestinal cancers showing a trend to higher incidence [1]. For advanced disease combinatory chemotherapy remains the mainstay of clinical management but outcomes are disappointing and prompt pursuit of alternative treatment modalities. Generally speaking, immunotherapy and especially immune checkpoint-directed therapy is now revolutionizing the management of oncological disease, an endeavor even awarded the Nobel prize [2]. Cancers are antigenic and evoke immunological responses, but can escape the resulting tumor destruction through a variety of mechanisms including upregulation of so-called checkpoints: inhibitory elements to limit self-damaging autoimmunity. By counteracting these inhibitory signals, the cancer can be combatted. Such strategies have proven successful for treatment options in a range of solid tumors, including melanoma [3,4,5] and cancer of the lung [6,7,8]. Unfortunately, results for immune checkpoint inhibitors for treating gastrointestinal cancers have proven disappointing, urging exploration of strategies that might augment the potential of such drugs that are depending on the a priori presence of immune responses, as they do not initiate but enhance these [9]. 

An obvious strategy to improve anti-cancer immunity apart from checkpoint inhibition is vaccination. Vaccinating is the act of injecting a pathogen or foreign protein with the goal to induce antigen specific immune responses and immunological memory. Vaccination relies on the action of professional antigen presenting cells (APCs) such as dendritic cells (DCs) that via presentation of antigens on MHC class I and MHC class II initiate CD8+ cytotoxic T cell (CTL) and CD4+ T helper (Th) responses, respectively. The latter are required to obtain long-lived and effective CTL responses [10,11]. 

Because of lack of efficacy by immune checkpoint inhibitors in gastrointestinal cancers, vaccination is of high interest to be explored to initiate responses which can then be later on enhanced by add-on treatment with immune checkpoint inhibition. Design of vaccination strategies is complicated by the complex tumor microenvironment (TME) and other characteristics like mutational load and expression of tumor antigens, which are largely unique to various types of tumors and may vary even within tumors. This is not different for gastrointestinal cancers.

As a consequence of the expression of embryonic or germline antigens, or because of genomic alterations leading to neoantigens, cancers can become immunogenic. Neoantigen load shows substantial variation between different forms of cancer and correlates to a certain extent with the success of checkpoint-directed immunotherapy [12]. Concordantly, mismatch repair deficient gastrointestinal (e.g., colorectal and pancreatic) cancers that bear many mutations are more responsive to checkpoint-directed therapies [13]. However, high neoantigen levels do not correlate with survival for pancreatic- and liver cancer per se [14,15,16]. Yet also for these cancers it is rational to assume that stimulating cancer-specific immune responses will be associated with better outcomes. However, in these situations, optimal exploitation of the available antigenic targets and combination therapies that overcome tumor specific suppressive mechanism are likely required. 

Nowadays we discriminate between two types of vaccination. Prophylactic (preventive) vaccines and therapeutic vaccines. A few examples can be given of prophylactic vaccines that are very effective in preventing cancer, the human papilloma virus (HPV) vaccine, preventing cervical cancer and the hepatitis B virus (HBV) vaccine, preventing liver cancer [17,18,19]. However, for established disease, these vaccines are also not effective because they typically induce humoral rather than cellular responses. In the present manuscript we shall overview the most important therapeutic cancer vaccine forms, elude on non-immune related cancer therapies that may trigger systemic immunity as a side effect, and will discuss how these therapies mechanistically offer potential for combination with other forms of immunotherapy to find opportunities for treatment of gastrointestinal cancers.

## 2. The Ideal Anti-Tumor Immune Response and the Limitation of Vaccination

A long-established cancer immune-editing theory describes the interplay between a cancer and the immune system, encompassing three phases: elimination, equilibrium and escape (the three E’s) [20]. According to this view, initially the immune system can control cancer cells (*elimination*), a process also termed immune surveillance. However, certain clones of malignant cells missed by the immune system (e.g., due to a non-immunogenic phenotype), escape the elimination phase (*equilibrium*). The clones that survive are then subject to immune pressure driven (epi)genetic editing, which ultimately leads to *escape* of the tumor from immune control [21,22]. In cancers these three phases can occur simultaneously in patients. Immune checkpoint blockade (ICB) has the potential to shift the balance to elimination and equilibrium. Importantly, low-fitness neoantigens may be leveraged by vaccination, i.e., marginal antigens in the immunosuppressive environment of a cancer that do not provoke effective immunity, when triggered by vaccination may confer effective anti-cancer responses [23].

Suppressive mechanisms however may limit the effect of vaccination. Tumors actively keep the immune system at bay by shielding themselves from the outside with a thick stroma or fibrotic shell [24], an anti-inflammatory microenvironment containing immune suppressive cells like M2-macrohpages [25], regulatory T cells [26], myeloid derived suppressor cells (MDSCs) [27], or by utilizing immune pathways like the PD1-PDL1 axis to suppress responses [28,29,30]. For gastrointestinal cancers these anti-cancer immune suppressing mechanisms show substantial redundancy as in situ approaches to enhance immune system activity through local application of non-relevant vaccines (e.g., anti-rotaviral vaccines or anti-yellow fever vaccines) only generate local immune responses to cancer when combined with ICB [31,32]. Hence, overcoming the resistance to immune response development in gastrointestinal cancer, requires targeting multiple pathways. 

How this may be achieved is outlined in the canonical tumor immunity cycle of Chen and Mellman. Here, the cancer immune response is described as an ongoing cycle of tumor cell killing and subsequent initiation of new responses which may combat the adaptation of tumors [33]. To prevent tumor escape, continuous killing of tumor cells is required to trigger responses also against novel antigens expressed by escaping tumor cells. Vaccination may trigger an initial “therapy-induced hit”, further releasing antigens and danger signals kick-starting the cycle. Ideally this therapy-induced hit should also alter the anti-inflammatory environment in the tumor to a favorable pro-inflammatory environment, and facilitate the influx of novel T cell clones recognizing antigens beyond those starting the response and thereby create a snowball effect leading to a broad T cell repertoire. [34,35]

**Figure 1:**
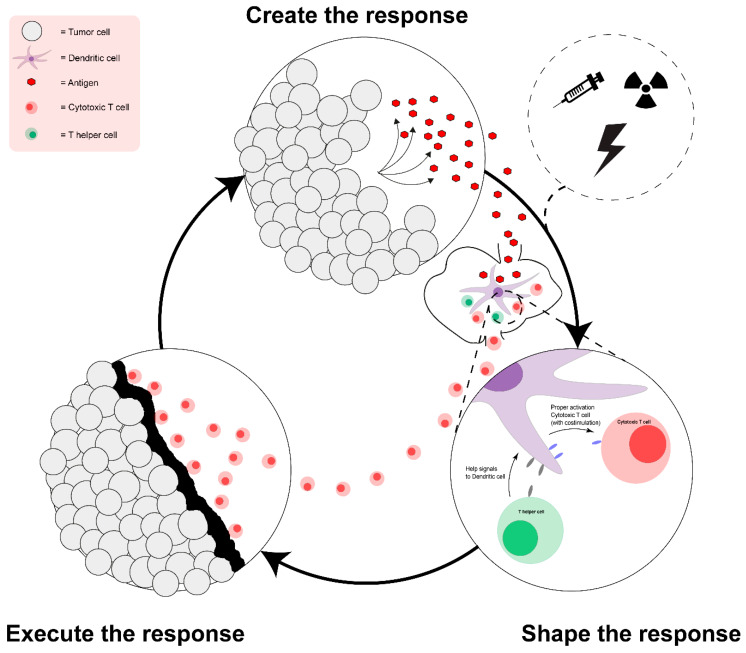
A simple representation of an anti-tumor immune response with integration of (in situ) vaccination. In case of naturally arising anti-cancer immunity, antigens are released from the tumor, creating the response. Antigens end up in the lymph node and are presented on dendritic cells, to T helper cells and cytotoxic T cells. T helper cells give help signals to dendritic cells resulting in enhanced costimulation for cytotoxic T cells, shaping the response. Activated T cells will migrate to the tumor and kill the tumor cells, executing the response. However, T cells at the tumor site may encounter a harsh microenvironment which often starts with a physical barrier. By killing the tumor cells new antigens are released and the cycle can continue. In of the absence of naturally arising immunity, (in situ) vaccines can be used to kick start the response.

To obtain an effective immune response in cancer patients three steps are generally thought to be required (Figure 1): (1) Creation of the response: under certain circumstances a tumor specific CTL response might already exist, but in many cases, there is either no response or the response is ineffective. Absence of a response is likely present in immune desert tumors that encompass a minor but significant part of gastric, colorectal and pancreatic cancers [36]. Although for some tumors antigenic targets may have been largely absent (restricting vaccination opportunity), for others responses may have lacked because tumor specific antigens did not (yet) reach APCs/DCs or the APC triggered response was subsequently not properly shaped. The treatment modalities outlined in Table 1 and Table 2 mostly can support this very first step, the initiation of CTL and Th responses. Initiation can be achieved through conventional vaccination, with manually selected target antigens, or through in situ vaccination, releasing antigen via immunogenic cell death (ICD) to initiate the response. The latter option has the benefit that this is not limited to a set of patients expressing a specific selected antigen. (2) Shaping of the response, during T cell priming by APCs in the lymph node (LN), the costimulatory signals received by the T cells are detrimental for the efficacy of the eventual response. These signals are provided by DCs activated and maturated by danger signals and/ or by contact with activated MHC class II primed Th cells. It is pivotal for their efficacy that CTLs receive the correct ‘help’ signals during priming in the lymph node. The most prominent example is the CD28-CD80/86 axis, but other pathways like the Th supported CD40-CD40 Ligand or CD27-CD70 axes have also been proven essential for the ability of CTLs to migrate towards, infiltrate in and ultimately to kill tumors [10,11]. As such, lack of help may contribute to the immune exclusion phenotype which marks a large fraction of gastric, colorectal and pancreatic cancers [36]. Furthermore, the absence of appropriate costimulatory and help signals can contribute to the exhausted or dysfunctional T cell phenotype often observed in cancer [10,11,37]. These signals are also a point of intervention for immunotherapy. Examples are blocking antibodies for CTLA-4 (ipilimumab; a competitive inhibitor of CD28) or agonists for CD40 that each may enhance or direct the shape of the response [38,39]. Combination of such drugs with vaccination could thus enhance the potency of the vaccine-induced response. (3) Executing the response, after the adaptive response has been established, fully primed and armed, T cells need to infiltrate the tumor and kill the tumor cells. Only then T cells will start a new cycle, tailoring immunity to the evolving cancer until the tumor is eradicated and memory is established, thus preventing also future growth of the tumor. Execution of CTL responses, however, are in many cancers, including gastrointestinal cancers, locally suppressed by an array of suppressive molecules and cells, such as PD1-PD-L1 or MDSCs respectively [40,41]. In addition, cancer specific suppressive mechanism may prevent immune effector function and thus limit the effect of vaccination. Pancreatic ductal adenocarcinoma for example is notorious for its fibrotic immune suppressive TME that may need to be tackled (e.g., by focal adhesion kinase inhibitors) for immune responses to take effect [42,43,44]. In colorectal cancer aberrant WNT/β-catenin signaling shapes the TME and can render these tumors unresponsive to ICB and may therefore require specific attention when combined with vaccination or immunotherapy [45]. A suppressive TME may especially impair the effect of conventional vaccines that start the response outside the tumor and do not much to improve the local environment. On the other hand, this might be an extra opportunity for in situ vaccines, that by definition also affect the local environment and, by disruption of the tissue or the release of chemotactic factors, might enable infiltration of immune cells [46]. How do presently employed strategies relate to the above-described idealized scenario?

## 3. Conventional Vaccines

The first cancer vaccine exploiting the immune system for cancer treatment, named ‘Provenge’ or “Sipuleucel-T”, was an infusion of DCs, isolated from the patient and loaded with a specific antigen ex-vivo [93]. Over the years many more vaccine forms/platforms have been developed aiming to bypass the first step in the cycle (Figure 1), to create an immunological response by offering the antigen in various forms, processed or unprocessed, to the patient. Various vaccine platforms deliver antigens in many forms and complexities ranging from tumor lysates to whole proteins, protein encoding mRNA, protein fragments or synthetic long peptides (SLPs) and to finally 9-11 AA short peptides of the minimal MHC class I binding epitope (Table 1). Although vaccines thus far have yielded immunological and some clinical effects, their clinical efficacy is still disappointing [64,94,95,96]. The use of suboptimal vaccine platforms and of low immunogenic vaccine target antigens (e.g., overexpressed self-antigens) together with a suppressive tumor microenvironment is held responsible, as has recently been extensively reviewed elsewhere [97].

We will first briefly go over the main vaccine platforms and discuss their ability to create or shape response and to what extent they may need additional support. Because danger signals are crucial for the shaping of a response, conventional vaccines are often combined with adjuvants. Especially when low immunogenic self-antigens are targeted, such as overexpressed tumor antigens for which central tolerance exists, adjuvants are likely very important. The need for adjuvants and the type of adjuvants used may also differs per vaccine platform as will be touched upon below. We will, however, not discuss the various types of adjuvants in detail as there are some recent excellent reviews on this matter [98,99].

### 3.1. Peptide Vaccine

Peptide vaccines exist in a short or long format, are generally stable, safe and can be used of the shelf for common tumor (specific) antigens or in a personalized fashion. Furthermore, peptide vaccines are cheap and easy to produce (Table 1). However, for personalization, genetic analysis of the tumor is required which may delay treatment and is not always possible to perform (for instance in inoperable pancreatic cancer). Short peptides (<15 amino acids (AA)) are convenient because of their ability to directly bind MHC, but short peptides are MHC subtype restricted and may also induce tolerance or on-target off- tumor toxicity by binding to MHC on non-professional APCs [38,51,100]. Synthetic long peptides (SLPs; ≈15–40 AA) in contrast, need to be processed by professional APCs rendering these safer and less tolerogenic and non-MHC restricted. For peptide vaccines obtaining sufficient MHC-epitope complexes for the creation of a response is easier than for whole protein-based vaccines [68]. Furthermore, SLPs can also provide MHC class II epitopes facilitating activation of CD4 T helper cells and have a high epitope concentration. Peptide vaccines may benefit from Th-skewing adjuvants, which can also be conjugated to the peptide and can further help shaping the response [50,101]. Combinations of peptide vaccines with forms of immune therapy that aid in the later stages of the response are obvious and good options, as long as sufficient T cells are induced and able to not only migrate to, but also infiltrate the tumor.

In clinical practice peptide vaccines, have been and are used treat premalignant advanced or recurrent HPV16-induced gynecological carcinoma but also a multitude of cancers targeting cancer (neo)antigens [48,49]. Targeting HPV with SLPs may be of high interest also for the treatment of HPV related esophageal cancer [102]. Especially SLP vaccines have shown promising results with respect to the creation of both CTL and Th responses that also correlated with clinical effects. In premalignant HPV lesions more than 50% of patients showed a complete or partial response (i.e., regression of lesions) upon SLP vaccination [103]. In malignant disease responses were less overt. Although, immunological responses induced by the vaccine were observed in a majority of tested patients, no regression of tumors nor prevention of progressive disease was observed likely because T cell were impaired in the execution phase by immune suppression [104]. To lift suppression, combination of SLPs vaccines, with low-dose chemotherapy to kill suppressive myeloid cells, was shown to improve T cell responses [105,106]. Furthermore, it was found that the tumoricidal effects of PD-1 inhibition (with nivolumab) may be enhanced by combining it with an SLP vaccine. These encouraging results were, however, obtained in a phase II single-arm study and need to be confirmed through a randomized control trial before changes in clinical practice are indicated [107]. Recently, also a personalized neoantigen-based SLP vaccine showed highly promising immunological (i.e., Th and CTL) and clinical responses with and without additional ICB therapy in metastatic melanoma [108]. In an alternative approach, recently a Phase I immunotherapy trial with two chimeric HER-2 (commonly over-activated in gastrointestinal cancer) B-Cell long peptide vaccines were tested in solid tumors including gastrointestinal tumors and showed anti-tumor activity with a very acceptable side effect profile. This study indicates that long peptides may be even more versatile, triggering not only tumor directed cellular but also humoral immune responses [48]. It should prove very interesting to combine such approaches with immune checkpoint-directed therapy and assess the potential to control gastrointestinal cancer refractory to ICB monotherapy.

### 3.2. Genetic Vaccine

RNA and DNA vaccines are genetic vaccines. Genetic vaccines rely on the concept that DNA or RNA encoding for antigens are transfected into cells and serve as a template for proteins synthesis, maintaining the native structure of the protein. Material from transfected cells may engage the MHC class I and II pathways in DCs/ APCs or DCs can be directly transfected themselves and present peptides on MHC I via the endogenous route of antigen presentation. Genetic vaccines may thus theoretically induce humoral and both CD8 cytotoxic T cell responses and CD4 T helper cell responses although the extent of each may vary depending on the dominant target cell of a specific genetic vaccine. [55,109] Genetic vaccines are relatively cheap and simple to synthesize. They are safe and highly flexible and a broad range of antigenic targets can be selected with this technique. However, genetic vaccines may be limited in immunogenicity and the antigen levels obtained are more variable and harder to control than for peptide vaccines. Yet, the genetic vaccine has come a long way with many optimizations in, e.g., codon optimization, novel plasmid vectors, vector boosting regimens and more. [56] Although genetic vaccines have intrinsic adjuvant properties by binding to pattern recognition molecules recognizing nucleic acids, this may not necessarily aid their effect as it induces and antiviral state, abolishing antigen translation [109]. Rather, for optimal efficacy an adjuvant effect may need to be pursued after genetic vaccine induced antigen production. This can be achieved for example by co-expression of immune activating proteins (e.g., CD40L, CD70) or cytokines (e.g., IL12) [109]. Alternatively, potential for combination with other forms of immune therapy might also lie in the priming and shaping phase. For example, therapeutic compounds targeting the APCs for enhanced immunogenicity like CD40 agonists or other T cell activators in clinical development to aid in the shaping of the response. 

DNA vaccination has been clinically tested in HPV related neoplasia and a multitude of cancers. On precancerous HPV lesions the vaccine had beneficial effects causing histopathological regression in a significant number of patients. [110] However, clinical trials with DNA vaccines in more established diseases like melanoma, prostate-, colorectal- or breast cancer disappointed in terms of therapeutic outcome, despite the immunological responses induced. [53,54,57,111,112,113,114,115,116,117] Yet, these results pave the way for combinations with therapies to lift the suppressive mechanisms of the tumor. Additionally, mRNA vaccines have been applied to many different cancers and have shown immunogenicity and some clinical responses [109]. Of special interest is a recent clinical study on the vaccination of 13 late stage melanoma patients with mRNA encoding mutated parts of proteins (27AA with the mutation in the middle; 10 potential immunogenic mutations per patient) that resulted in T cell responses against multiple neo-epitopes in all patients (mostly Th but also CTL). Despite low patients numbers this study also showed promising clinical effects including a complete response in one patient receiving the vaccine combined with PD-1 blockade [118].

For many approaches, antigen selection remains a bottleneck. The most obvious way to address this is combining genetic analysis of the cancer and patient HLA phenotype with prediction tools that identify promising candidates. Now that many centers are building molecular precision medicine pipelines for drug selection in gastrointestinal cancer, it is also becoming feasible to use the infrastructure for selecting epitopes suitable for personalized genetic vaccines, which in combination with ICB therapy may prove exceedingly useful. 

### 3.3. Tumor Cell Vaccine

Tumor cell vaccines are whole-cell vaccines consisting of inactivated allogeneic tumor cell lines or of autologous tumor cells. They contain characterized, but also uncharacterized, tumor antigens which lie at the basis of inducing the immune response. Examples are Canvaxin and GVAX [119]. GVAX is a tumor cell vaccine where the origin of tumor cells can be autologous or allogeneic (can be given to a broader target population). Because the vaccine consists of ‘whole protein’, it will contain Th and CTL epitopes. In GVAX, the tumor cells are engineered to express granulocyte macrophage colony stimulating factor (GM-CSF). In mice genetically modified tumor cells engineered to express cytokines like interleukin 2 (IL-2), interferon gamma (IFN-y) or GM-CSF can be rejected and can induce systemic immunity. Subsequent characterizations of the induced immune response revealed a local influx of immature dividing monocytes, granulocytes and activated lymphocytes at the injection site. Moreover, paracortal hyperplasia was observed at the draining lymph node. Most of this preclinical work was done in mouse models of melanoma but was also extended to renal cell carcinoma, colon carcinoma and fibrosarcoma models [119,120]

Although these preclinical results were promising, the clinical efficacy of GVAX was thus far limited. Studies have mostly been performed in (but not limited to) prostate cancer, pancreatic cancer and colorectal cancer [58,60,61,121]. Immunologically, Th cells have been demonstrated to be induced upon treatment with GVAX, however, these studies often included combination of GVAX with checkpoint inhibitors like ipilimumab [60]. This complicates our understanding of the sole effect of GVAX on the adaptive immune response. Additionally, due to allogeneic HLA, the vaccine might be rejected and may not induce an effective anti-tumor immune response. GVAX-ICB combinations are currently pursued further in the clinic. [58] Interestingly, one of the biomarkers that was found associated with survival in pancreatic cancer following GVAX combined with ipilimumab was a diversification of the T cell receptor (TCR) repertoire [58,121]. Although ipilimumab has this effect already by itself, diversification was most clear upon co-treatment with GVAX [58,61,121].

### 3.4. DC-Vaccine

Dendritic cells are considered the most important professional APC crucial for the initiation of any adaptive response [122]. They are very efficient in the phagocytosis of antigens, and subsequently process these and load derived peptides on MHC class II. In addition, DCs excel in the cross presentation of incoming antigens on MHC class I to T cells. DCs also provide the necessary costimulation to T cells for proper activation and function. Finally, they can secrete cytokines that further shape T cell function. In vivo different DC subtypes can be discriminated (i.e., myeloid DC1, DC2, plasmacytoid DC and inflammatory monocyte derived DC) that differ in function [123]. Of particular interest are the rare subset of DC1 that are thought to excel in cross presentation and in mice have been demonstrated to be crucial to the activation of naïve T cells and are thought to transfer help signals to CD8 cytotoxic T cells through CD4 T helper cells [124,125,126]. DCs can be loaded with antigens and activated ex-vivo and be given to a patient as a therapy [15,62,63,127,128,129]. For loading of vaccine DCs all the aforementioned forms of antigens can be used (i.e., short and long peptides, DNA, RNA and tumor lysates). For DC vaccination monocyte derived DCs (moDCs) have been popular because they can be easily differentiated ex-vivo from monocytes that can be obtained in large numbers through leukapheresis. Current thought is, however, that moDCs are not the most optimal DC for vaccination [66,130]. Primary DC subsets, which can only be harvested in lower numbers from patients, may be more effective and have recently also been used for vaccination with promising results. The DC type used, the antigen loaded and the activation method used together likely greatly determine the ability of the DC to create and shape a response. Efforts are currently directed at the exploitation of primary DC subsets including rare DC1 for vaccination and at optimizing DC loading and activation [130].

Although DC vaccination is time and resource consuming, antigen loading and DC activation can be well controlled and monitored which is less for other cancer vaccine platforms. DC therapy has been proven to be safe in the clinic and preliminary data deems it efficacious, triggering both Th and CTL responses and also yielding some clinical responses [66]. Currently DC vaccines are tested in several advanced phase II/III trials including gastrointestinal cancers [69]. For DC therapy, however, use as a stand-alone therapy has thus far been disappointing despite their proven ability to trigger T cells [67]. DC vaccines very likely require support of T cells in the execution phase for clinical effect. Concordantly, many trials with combinations of DC vaccines with checkpoint inhibitors like PD-1/ PD-L1 and CTLA-4 inhibitors are ongoing [65,69,131].

## 4. In Situ Vaccines

Besides these conventional vaccination strategies, there are also several other therapies that can have an in situ vaccine effect which initial purpose was not to generate immunological memory or an immune response at all [71,132,133]. These treatment modalities can cause the release of antigen and thereby can have a vaccine-effect in situ, resulting in the induction of an immune response and the development of immunological memory [134]. Their strongest edge over most conventional vaccines is that screening of the patient for antigen-positivity is not needed [72,85,135,136,137]. For these in situ vaccines the effectiveness of the resulting immune response depends on the expression of immunogenic antigens in the tumor at the time of treatment. The response will by definition be ‘personalized’, and the lack of need to screen for tumor antigen expression may save valuable time. Furthermore, in case of local treatment and induction of a systemic immune response, also metastasis might be targeted indirectly due to the partial antigenic similarity of the main tumor and the metastasized tumors (i.e., an abscopal effect) [73]. An overview of the most important therapies with a known in situ vaccination effect is given in Table 2. 

The concept of in situ vaccination comprises that the antigens causing the vaccine effect are already present in the tissue and are released upon therapy. Upon release these antigens are taken up by phagocytic cells and transported to the lymph node for the induction of specific, personalized adaptive immune responses [86]. In situ vaccination is thus an attractive form of personalized medicine as any tumor will have its own profile of tumor antigens and mutations that might form neoantigens (i.e., new antigens to be loaded on HLA-molecules). For treatments having an in situ vaccine effect, tumors do not necessarily have to be characterized before starting treatment, saving valuable time. Possible limitations of the in situ vaccination however, might be that antigens might not be present in such a concentration to allow effective antigen (cross)presentation and the creation of proper responses. Furthermore, as the antigens carrying the vaccine effect are not known it is difficult to monitor the response [74]. Lastly, antigen release following these treatments might not always be accompanied by sufficient danger signals to shape the response (and break tolerance in case of self-antigens). Especially in this scenario, responses following the in situ vaccination may benefit from immunotherapeutic agents that are designed to stimulate/initiate key mechanisms important to the shape and execution of an effective adaptive immune response [135,137]. Although many conventional cancer therapies used to treat gastrointestinal cancers may have an in situ vaccine effect, we will restrict our discussion to those most widespread used. 

### 4.1. Radiotherapy

Radiotherapy is still one of the most important treatment modalities for cancer and is also standard-of-care or at least a treatment option for many gastrointestinal cancers [138]. It causes radiation-induced cell death trough lethal DNA damage [139]. A secondary effect of radiotherapy is activation of the immune system as it leads to ICD of the tumor cells by ionizing radiation [81]. However, complications might arise due to destruction of not only cancerous tissue but also healthy tissue, inducing so called ‘late-effects’ that might even lead to an increased risk of getting cancer in a later stage of life due to the radiation. 

Radiotherapy not only releases antigens for uptake by APCs but may also provide cell death-associated danger signals (e.g., cell surface calreticulin, ATP, nucleic acids, HMGB1) important for DC activation and immune cell recruitment [140]. Thus, radiotherapy may be effective to create and shape the response. Apart from the activation of the immune system, effects of ionizing radiation are also seen in the tumor microenvironment for example on the vascular endothelium where factors involved in the recruitment of T cells were increased following radiotherapy [76,77,141,142,143]. It is important to note, however, that not every radiation dose has the same effect. In mice, the release of cell free DNA in the tumor was found to be compromised by the expression of DNA exonuclease following a single high radiation dose. This was thought to prevent the activation of the cGas-STING pathway and therefore prohibited immune cell activation. In this same study combination of radiotherapy with a CTLA-4 inhibitor, provides not only a local immune stimulatory effect but also an abscopal effect by the generation of systemic immunity [144]. Furthermore, in a mouse model of pancreatic cancer the induction of tumor specific memory cells by radiotherapy was enhanced by combination with a CD40 agonist [145].

One of the concerns of combining radiotherapy, as the inducer of the immune response, and ICB, removing the brakes from the immune response, is safety, especially as also self-antigens are released. Checkpoint inhibitors are known to have side effects, and when combined with each other, these might occur significantly more [146]. Combining radiotherapy with ICB, however, has thus far been reported to be safe and well tolerated [147,148,149].

In mice, anti-CTLA4 treatment predominantly inhibited regulatory T cells while radiation therapy enhanced the TCR repertoire of intratumoral T cells. When these therapies were combined, anti-CTLA4 promoted the expansion of T cells and radiation shaped the TCR repertoire of the expanded peripheral clones, thus these modalities synergized to create the response as well as to shape the response [149]. However, in patients this combination was less effective. Melanoma patients showing high PD-L1 expression in the tumor, when treated with ionizing radiation together with anti-CTLA4, developed T cells with an exhausted phenotype and the tumors progressed [149]. In lung cancer a case of clinical success of combination of RT with CTLA-4 was reported [150] and also combination with PD-1 blockade showed promising results [151]. In mice it was shown that in addition to ionizing radiation and anti-CTLA4, supplemented with PD-L1 blockade reversed T-cell exhaustion and aided in the execution of the response suggesting further combination of radiotherapy with multiple checkpoint inhibitors could be more effective [147,148,149] Although the combination with radiotherapy may be superior to just ICB, not all inhibitors have the same efficacy and are based on different mechanisms. More mechanistic insight is now required to make good combinations—optimally covering all three requirements depicted in Figure 1 [140].

### 4.2. Chemotherapy

Chemotherapy is extremely versatile and apart from stopping tumor proliferation directly it may also aid the generation of anti-tumor immunity. In general, it is used as a therapy to manage disease and treat lower grade cancers. [152] However, chemotherapy also holds potential to enable other therapies to become more efficacious in late stage cancer. Immunological effects of chemotherapy can be induction of ICD, releasing both danger signals and tumor antigens facilitating antigen presentation, induction of a cellular senescence program in tumor cells that alert the immune system by activation of natural killer (NK) cells and finally the inhibition of immune suppressive cell populations like regulatory T cells or myeloid derived suppressor cells (MDSCs). [78,82,83] Most important chemotherapeutics that lead to ICD are idarubicin, epirubicin, doxorubicin, mitoxantrone, oxaliplatin, bortezomib and cyclophosphamide [153]. These individual chemotherapeutic agents have been extensively discussed for their specific immune modulatory properties elsewhere [153,154,155]. It is important to note that the immunological effects of chemotherapeutic drugs might vary greatly. This is illustrated by differential ICD in response to the related chemotherapeutics cisplatin (no ICD) and oxaliplatin (ICD), both commonly used to treat gastrointestinal cancers [153,156]. Thus, not all chemotherapeutics may benefit similarly from a combination with immunotherapy. 

In general, combination of chemotherapy with checkpoint inhibitors (anti-CTLA4 or anti-PD-(L)1) is well tolerated. In various types of cancer like lung cancer and biliary tract cancer, the combination also seems to be superior compared to single immunotherapy or chemotherapy alone. [157,158,159,160]. Additionally, for pancreatic cancer the combination of gemcitabine chemotherapy with PD1 was well tolerated and holds promise [161]. 

Although combination of ICB with chemotherapy occurs very often in clinical trials, this is most often because it was a standard of care treatment for these patients rather than a rational choice based on the immunological effects of specific chemotherapeutic agents. However, there are several exceptions that are explicitly meant to exploit the immune stimulating actions of ICD-inducing chemotherapeutics [153,162]. One of these is a recent multi-arm phase II study comparing the combination of various ICD and non-ICD inducing chemotherapeutics with PD-1 inhibitor nivolumab [163]. Results confirmed the superiority of combining ICB with ICD-inducing chemotherapy (in this case doxorubicin).

### 4.3. Oncolytic Viruses

Oncolytic viruses (OV) have been discovered by accident in patients from cases that experienced tumor reduction after contracting a natural viral infection [91,92]. OV based on naturally replicating viruses are selective for tumor cells in particular. These viruses exploit the fact that tumor cells, in order to attain features beneficial for uncontrolled growth, trade in some basic biological processes, one if which is the innate response mechanism to viral infection. Because this is lacking in tumor cells, oncolytic viruses can specifically infect the tumor and cause cell death. Although various viruses have been tested for this purpose, of particular interest are two recent studies in mice, demonstrating oncolytic effects after intratumoral vaccination with common prophylactic vaccines based on attenuated viruses (i.e., yellow fever and rotavirus) [31,32]. Another class of oncolytic viruses is formed by viruses genetically modified to target and kill a tumor [92]. Although it was thought that direct cell killing by the virus was responsible for tumor control/regression, evidence is accumulating that systemic immunity that can originate from this killing (an in situ vaccine affect) is also very important. Like radiotherapy and chemotherapy, OVs can cause ICD, releasing antigen and promote a local pro-inflammatory environment, leading to an adaptive immune response [89]. Additionally, recombinant OVs are being tested in the clinic carrying various tumor antigens, using the OV simultaneously as a viral vector [87]. In comparison to radiotherapy and chemotherapy, the experience with the combination of oncolytic viruses and ICB is still in its infancy [31,92]. Clinical trials combining several different form of OVs (including recombinant OVs carrying various types of antigen) with PD1/PD-L1-, CTLA-4 inhibitors or other forms of immunotherapy are currently ongoing [164]. Pioneering clinical results have been obtained in melanoma where response rates with a genetically modified GM-CSF expressing herpes simplex based OV (T-VEC) in the presence of CTLA-4 or PD-1 blockade were promising and even improved in patients treated with OV combination therapy compared to anti-CTLA4 alone [90,165,166]. 

## 5. Conclusions

To obtain the best therapy combination of immune stimulatory approaches that create and shape an effective adaptive anti-tumor response and also support this response optimally in the execution phase, it is important to characterize the immune status of a patient (-population) [167]. In case of evidence of an ongoing active immune response, ICB can be considered as a stand-alone treatment. However, in most cases there is no proper adaptive immune response against the tumor. Vaccination offers the possibility to create a response, inducing T cells, but especially for gastrointestinal cancers additional support of the response through checkpoint inhibitors may prove essential. There are many forms of vaccination and therapies with an in situ vaccine effect, as were discussed in this review. Therapies with in situ vaccination effects provide considerable opportunities, as they do not depend on the characterization of tumor antigens or vaccine design/ manufacturing and may also disrupt the TME which greatly limits immune resolution of many gastrointestinal cancers. Novel therapies like OVs are of high interest but also more common therapies like radiotherapy and chemotherapy that are already part of routine clinical practice may prove exceedingly useful in this respect. To find more effective therapies for ICB resistant gastrointestinal cancers, it seems worthwhile to evaluate and investigate immunological effects of non-immune standard-of-care treatments. A direction might be to identify and investigate intrinsic features of gastrointestinal cancers like composition of the TME. A tumor devoid of T cells, or populated with mainly exhausted terminally differentiated non-responding T cells might be in need of a new immune response cycle. Such tumors are ideal candidates for (in situ) vaccination. For tumors with a low mutational load targeted therapies like peptide vaccines could be utilized to induce or enhance CTL responses. However, with a higher mutational load and/or higher immunogenic antigen presentation radiotherapy or chemotherapy may be the preferred way to get that initial release of antigens. 

However, immune regulatory mechanisms may still be in place that will prevent antitumor immunity. Identifying these mechanisms in a personalized manner can aid in the selection of immune checkpoint inhibitors (or alternative therapies) to combine with vaccines to give that last push to shift the equilibrium to tumor cell killing and promote remission in patients. For example, a patient harboring only little or dysfunctional/helpless CTLs could be treated with a vaccine and subsequently with PD(L)1 inhibition to rescue effector function at the tumor site. 

To make effective combinations we now need more detailed insight into the power and mechanism of each (in situ) vaccine form to create and shape the response and also more knowledge on the timing of the created response. Furthermore, we need to know what essential properties induced T cells may lack, so specific signals or blockages can be provided to fix these shortcomings. [168] Only then can we provide the best combination immunotherapy at the right moment. In the near future the repertoire of checkpoint inhibitor options will expand. Many new forms of such therapy are currently in clinical development including blockade of LAG3, TIGIT, IDO, CD47, or TIM3, especially the latter may be of particular interest to combine with treatments causing ICD as it is a receptor for HMGB1 keeping this compound and associated DNA from triggering TLRs [169,170]. Additionally, several immune stimulators are currently clinically evaluated and may be used to enhance the shaping of adaptive responses following (in situ) vaccination (e.g., activators of OX40, GITR) [169]. Moreover, for combinations of (in situ) vaccine forms with these compounds we need more insight into the level at which vaccine-induced responses require support. Especially for in situ vaccines this may be challenging, as the nature of the antigens driving the vaccine effect is not known. State of the art analysis techniques may give answers. For example, immune responses in these patients could be followed by non-invasive tests like a screening for the TCR repertoire diversity in blood before and after initial therapy and by subsequently tracing back the phenotype of cells carrying prevalent TCR using single cell sequencing. [171] By verifying induction of an immune response after a first ‘therapeutic hit’, as described in Figure 1 (create the response), this can be followed-up by treatment modalities that support the effector cells and aid in executing the response, potentially leading to a superior treatment strategy against cancers in general and gastrointestinal cancers in particular.

## Figures and Tables

**Table 1:** Overview of conventional cancer vaccines with pros and cons.

Therapy	Pros	Cons	References
Peptide vaccines	Cheap, easy to produce Long peptides: Th and CTL epitopes, not HLA-restricted Personalized (neo-antigens) and semi-personalized (peptide “warehouse” for prevalent tumor antigens) High epitope concentration	Short peptides: no or less Th epitopes cells Restricted to selected epitopes/antigens Short peptides: HLA-restricted Poor immunogenicity (need adjuvants)	[47,48,49,50,51]
Genetic vaccines	Native sequence of protein Induce humoral and cellular response Personalized possible Th and CTL epitopes Cheap, easy to produce	Poor immunogenicity (needs adjuvants)	[52,53,54,55,56,57]
Tumor cell vaccines	Contains characterized and uncharacterized tumor antigens Th and CTL epitopes Allogeneic vaccine can be given, broader target population	Poor clinical efficacy Self/ normal proteins in the vaccine pose toxicity risk Possibility of release immunosuppressive cytokines Rejection of vaccine because of allogeneic HLA	[58,59,60,61,62,63]
Dendritic cell vaccines	Measurable antigen presentation efficiency and DC maturation Th and CTL epitopes	Not fully matured DCs/ tumor impaired DCs may induce tolerance Logistically challenging Costly, labor intensive	[64,65,66,67,68,69,70]

**Table 2:** Overview of in situ cancer vaccines with pros and cons.

Therapy	Pros	Cons	References
**Radiotherapy**	Depending on dose, can induce immunogenic cell death Can release uncharacterized/ personal tumor antigens Easy to combine with immune checkpoint inhibitors	Will cause ‘late effects’ Elevated risk of cancer due to treatment Destruction of healthy tissue	[46,71,72,73,74,75,76,77]
**Chemotherapy**	Can cause immunogenic cell death depending on the compound Can suppress specific types of immune suppressive cell populations Easy to combine with immune checkpoint inhibitors Will release uncharacterized/ personal tumor antigens	Overall toxicity Not all chemotherapeutic compounds have the favored immunogenic effect Destruction of healthy cells	[78,79,80,81,82,83,84]
**Oncolytic virus**	(Engineered to) Specifically target tumor cell Can Cause immunogenic cell death Will release uncharacterized/ personal tumor antigens Easy to combine with immune checkpoint inhibitors Can be engineered to express a tumor antigen or cytokines to modify the tumor micro environment	Anti-viral response can neutralizing the therapy, shortening the window of opportunity, Specialized facilities to monitor patients due to safety concerns	[32,85,86,87,88,89,90,91,92]
